# Single-cell transcriptomics identifies fibroblast associated immune heterogeneity and prognostic signatures in bladder cancer

**DOI:** 10.1038/s41598-026-38219-x

**Published:** 2026-02-03

**Authors:** Xiaojuan Tang, Ling Liu, Min Gao, Peng Duan, Sheng Li, Zilong Yuan, Qiang Xia, Lei Xi, Yan Tan

**Affiliations:** 1 Department of Radiology, Renmin Hospital, Hubei University of Medicine, Shiyan, 442000 Hubei P.R. China; 2 Department of Andrology, Renmin Hospital, Hubei University of Medicine, Shiyan, 442000 Hubei P.R. China; 3 Department of Anesthesiology, Renmin Hospital, Hubei University of Medicine, Shiyan, 442000 Hubei P.R. China; 4 Hubei Key Laboratory of Embryonic Stem Cell Research, School of Basic Medical Sciences, Hubei University of Medicine, No. 30 Renmin South Road, Shiyan, 442000 Hubei China; 5 Key Laboratory of Zebrafish Modeling and Drug Screening for Human Diseases of Xiangyang City, Department of Obstetrics and Gynaecology, Xiangyang No. 1 People’s Hospital, Hubei University of Medicine, Xiangyang, 441000 Hubei China; 6 Department of Radiology, Hubei Cancer Hospital, Tongji Medical College, Huazhong University of Science and Technology, No 116 Zhuodaoquan South Load, Hongshan District, Wuhan, 430000 Hubei China; 7 Biomedical Engineering College, Hubei University of Medicine, No. 30 Renmin South Road, Shiyan, 442000 Hubei China

**Keywords:** Bladder cancer, Single-cell RNA sequencing, Fibroblast marker, Immune microenvironment, Prognostic biomarkers, Biomarkers, Cancer, Computational biology and bioinformatics, Oncology

## Abstract

The immune microenvironment and prognosis of bladder cancer (BLCA) remain ongoing challenges in its treatment. This study aimed to establish predictive prognostic indicators and investigate the immune microenvironment to enhance clinical treatment strategies. A single-cell transcriptional atlas was constructed using single-cell RNA-seq data from patients with bladder cancer, focusing on fibroblast-related gene expression, intercellular communication, metabolic pathways inferred by single-cell flux estimation analysis, and transcription factor networks. Fibroblast-associated prognostic gene signatures were validated using data from The Cancer Genome Atlas, and a prognostic model was developed to stratify patients with bladder cancer into high- and low-risk groups. Analysis of three para-carcinoma single-cell samples revealed the presence of 3,603 fibroblasts and 500 fibroblast-associated marker genes. Notably, key fibroblast-specific transcription factors, including MAF, TWIST1, and TCF21, were identified through SCENIC analysis. The incorporation of comprehensive RNA sequencing data enabled the discovery of prognostic markers associated with fibroblasts. Using this classification model, patient survival could be stratified into high- and low-risk categories based on the model. The results of our study highlight the prognostic genetic signatures associated with the fibroblast component of the immune microenvironment in BLCA, offering preliminary insights into prognostic assessment and potential therapeutic implications.

**Supplementary Information:**

The online version contains supplementary material available at 10.1038/s41598-026-38219-x.

## Introduction

Bladder cancer (BLCA) is recognised as the second most aggressive tumour within the urogenital system globally, with an estimated 430,000 new cases and more than 165,000 fatalities attributed to BLCA each year^1,2^. Both the incidence and mortality rates of BLCA are increasing. In 2018, the global age-standardised incidence rate was approximately 9.6 per 100,000 men and 2.4 per 100,000 women, according to data on new cancer cases worldwide^3^. The high incidence and complex treatment protocols associated with BLCA pose a substantial challenge to health care within the field of oncology^4^. BLCA displays significant clinical variability, which can be difficult to detect in its early stages, leading to delayed diagnoses and poorer prognoses^5^. The tumour immune microenvironment plays a crucial role in influencing the progression and complexity of BLCA, functioning as a multifaceted system of interacting cellular components that may either inhibit or promote tumour growth^6–8^.

In the context of the tumour microenvironment (TME), cancer-associated fibroblasts (CAFs) are recognised as pivotal contributors to the progression of BLCA^9,10^. Once regarded primarily as structural components, recent investigations have highlighted their critical role in orchestrating immune responses^11–13^. Through mechanisms such as extracellular matrix (ECM) remodelling, secretion of tumour-promoting factors, and modulation of the immune milieu, these cells foster an environment conducive to tumour proliferation and immune evasion^14,15^. Nevertheless, our understanding of the distinct functions and mechanisms of CAFs in BLCA remains limited. The lack of comprehensive research into how CAFs facilitate tumour progression and evade immune detection in BLCA underscores the urgent need for further investigation.

The introduction of single-cell sequencing has significantly transformed our understanding of the intricate cellular environment by examining individual cells within tumours^16^. This methodology has revealed the heterogeneity among fibroblasts and other cells within the TME, providing insights into their diverse functions and interactions^17^. By capturing the gene expression patterns of individual fibroblasts, single-cell sequencing allows us to discern their nuanced roles in cancer progression, including their involvement in treatment resistance and the promotion of tumour proliferation and metastasis^18,19^. Building on these insights, our study employs single-cell sequencing to investigate the immune microenvironment of BLCA, with a particular focus on the fibroblast population. Through this analysis, we aim to deepen our understanding of the transcriptional regulatory networks and metabolic processes of fibroblasts. Furthermore, we have developed a prognostic model based on fibroblast marker genes identified through analysis of The Cancer Genome Atlas (TCGA) data. This model integrates detailed single-cell analysis with broad genomic data to stratify patients into different risk groups.

In summary, investigating the immune microenvironment of BLCA and developing reliable prognostic models based on fibroblast marker genes are essential for elucidating the complexities of the disease and implementing personalised treatment strategies, ultimately enhancing clinical decision-making. As our understanding of BLCA deepens, we are better equipped to manage the disease and improve patient outcomes. Exploring the immune and genetic dimensions of BLCA is crucial for the development of more effective treatments^20^.

## Materials and methods

### Data sources and processing

An overview of the study design and analytical workflow is summarised in Supplementary Fig. 1. The bulk RNA sequencing (RNA-seq) data, corresponding clinical information, and single-nucleotide polymorphism mutation data for TCGA-BLCA were obtained from The Cancer Genome Atlas (TCGA) database, including 19 normal bladder tissue samples and 411 BLCA samples. After excluding samples with incomplete survival or clinical information, a total of 406 patients with BLCA were retained for subsequent analyses. The single-cell RNA-seq (scRNA-seq) dataset GSE129845 was downloaded from the Gene Expression Omnibus (GEO) database (https://www.ncbi.nlm.nih.gov/). This dataset comprises scRNA-seq profiles of paracancerous tissues from three patients with BLCA. Sample integration was performed using the anchor-based integration method implemented in the Seurat R package^21^. Low-quality cells were removed by excluding cells with fewer than 200 detected genes, as well as genes expressed in three or fewer cells, to reduce technical noise and ensure data quality. Gene expression data of the retained high-quality cells were normalised and scaled using standard procedures implemented in Seurat, after which the top 2,000 highly variable genes were identified based on variance. Principal component analysis (PCA) was then conducted, and the top 20 principal components were selected for downstream analyses. Uniform manifold approximation and projection (UMAP) was applied for nonlinear dimensionality reduction and visualisation. Cell clusters were annotated manually based on marker genes curated from the CellMarker database and published literature^22,23^.

### Identification of cell clusters and functional enrichment analysis of marker genes

Marker genes for each cell cluster were identified using the FindAllMarkers function in Seurat with the parameters min.pct = 0.2 and only.pos = TRUE. Differential expression analysis was performed using the Wilcoxon rank-sum test, comparing each cluster against all remaining cells. Gene Ontology (GO) enrichment analysis of the top 500 marker genes for each cluster was conducted using the org.Hs.eg.db package in R, and results were visualised using the ClusterGVis package.

### Inference of regulons and activity

Gene regulatory network inference was performed using the single-cell regulatory network inference and clustering (SCENIC) framework. The SCENIC workflow consists of three main steps. First, a gene co-expression network was inferred using a tree-based algorithm to identify putative transcription factor (TF)–target gene relationships. Second, motif enrichment analysis was performed to refine the co-expression network and retain TF–target associations supported by the presence of TF-binding motifs, resulting in the identification of TF-centred regulons. Notably, SCENIC focuses on transcriptional activation and does not infer transcriptional repression. Third, regulon activity scores were calculated for each cell using the AUC ell algorithm, enabling the assessment of cell-specific TF activity patterns.

### Co-expression analysis using SEEK

To further assess the cell type relevance of the inferred regulons, search-based exploration of expression (SEEK) analysis was performed. The human version of SEEK was used to query publicly available GEO datasets for co-expression patterns between key transcription factors and their predicted downstream target genes identified from the SCENIC analysis. For each regulon, genes showing significant co-expression with the corresponding transcription factor across multiple independent datasets were considered to support the cell type specificity of the regulon. Statistical significance was evaluated using Fisher’s exact test, and regulons with significant enrichment were prioritised for downstream analyses.

### *CommPath* analysis

The *CommPath* R package is used for the analysis and inference of ligand–receptor interactions in scRNA-seq data^24^. In this study, we employed *CommPath* to investigate intercellular communication patterns within the BLCA immune microenvironment, with a particular focus on CAFs. *CommPath* was applied to identify the upstream and downstream signalling pathways associated with fibroblasts, as well as the communication chains that are notably activated within these cells. Subsequently, *pathHeatmap* was used to visually represent the activation pathways within each cell cluster.

### Single-cell flux estimation analysis (scFEA)

The scFEA model, a graph neural network used to infer cell-specific metabolic activity from scRNA-seq data, was implemented using the *scFEA* package in Python^25^. The model was trained on transcriptome data to predict metabolic flux at the cellular level, including the estimation of metabolic module activity and associated metabolite alterations. Through analysis of flux predictions, groups of cells or metabolic modules exhibiting similar metabolic profiles were identified. In our study, we highlighted the distinctive characteristics of metabolic modules displaying notable activity within fibroblasts.

### Least absolute shrinkage and selection operator (LASSO) regression analysis

Prognosis-associated genes were first identified in the TCGA-BLCA cohort. Genes that overlapped between fibroblast-related marker genes derived from single-cell analysis and prognosis-associated genes from bulk RNA-seq data were selected as candidate features for model construction. A least absolute shrinkage and selection operator (LASSO) Cox regression model was then applied to reduce feature dimensionality and identify the optimal prognostic gene signature. The LASSO analysis was performed using the glmnet package with ten-fold cross-validation to determine the optimal penalty parameter (lambda)^26^. Based on the selected gene coefficients, a risk score was calculated for each patient. To ensure that higher scores corresponded to higher risk, the final risk score was defined as the negative value of the linear predictor derived from the Cox model. Patients with BLCA were subsequently stratified into high-risk and low-risk groups using the median risk score as the cutoff. Kaplan –Meier survival curves were generated using the survival package, and risk distribution plots were visualised with the ggrisk package. Time-dependent receiver operating characteristic (ROC) curves at 1, 3, and 5 years were constructed using the timeROC package to evaluate the predictive performance of the risk model.

### Cox regression analysis and nomogram model construction

Univariate and multivariate Cox proportional hazards regression analyses were performed using the survival package to evaluate the prognostic value of the risk score and clinical variables in patients with BLCA. Variables with statistical significance in univariate analysis were subsequently incorporated into the multivariate Cox regression model to assess whether the risk score served as an independent prognostic factor. Clinical variables included age, sex, tumour stage, and tumour grade, where available. The results of Cox regression analyses were visualised using the survminer and forestplot packages. Based on the independent prognostic factors identified in the multivariate Cox analysis, a nomogram model was constructed using the rms package to estimate individual patient survival probabilities. Calibration curves were generated to assess the agreement between predicted and observed survival outcomes. All analyses were conducted using R software, and two-sided *P* values < 0.05 were considered statistically significant.

### Somatic mutation analysis

*Maftools* was used to analyse somatic variant data from BLCA samples stored in mutation annotation format (MAF). Tumour mutation burden (TMB) scores were calculated for each patient with BLCA, and the association between the risk score and TMB was examined. The TMB score was calculated as follows: (total mutations / total covered bases) × 10^6^.

### Pancarcinoma assay

This study examined the expression patterns of fibrillin-1 (*FBN1*), phosphotyrosine interaction domain-containing 1 (*PID1*), and proline/arginine-rich end leucine-rich repeat protein *(PRELP*) across multiple cancer types. Gene expression data were sourced from publicly accessible cancer genomic repositories, specifically TCGA and the Genotype-Tissue Expression (GTEx) project. Subsequent data preprocessing and normalisation procedures were performed using R software to ensure consistency and facilitate cross-dataset comparisons.

Differential expression analysis was employed to identify significant expression differences between cancerous and normal samples across various cancer types. Statistical tests were conducted to validate the significance of these differences, supporting the investigation of the potential roles and mechanistic implications of these genes in cancer development.

### Immunohistochemistry

Formalin-fixed, paraffin-embedded BLCA tissues and matched adjacent non-cancerous bladder tissues were obtained from three patients at Shiyan People’s Hospital, Hubei University of Medicine, Shiyan, China. These samples were used as representative specimens for immunohistochemical validation. This study was approved by the Institutional Ethics Committee (Approval No. SYRMY-2025-059), and written informed consent was obtained from all patients prior to sample collection. Through these assessments. Paraffin-embedded tissue Sect. (5 μm) were deparaffinised in xylene and rehydrated through a graded ethanol series. Antigen retrieval was performed by heating the sections in 0.1 M sodium citrate buffer (pH 6.0) using a microwave oven for 10 min, followed by cooling to room temperature. Endogenous peroxidase activity was blocked with 3% hydrogen peroxide in 85% methanol for 15 min. After blocking with 10% sheep serum in phosphate-buffered saline at 37 °C for 1 h, sections were incubated overnight at 4 °C with primary antibodies diluted in blocking buffer.

The primary antibodies used were rabbit anti-*FBN1* (A16677, ABclonal, China), rabbit anti-*PID1* (A13220, Abclonal, China), and rabbit anti-*PRELP* (A18573, Abclonal, China). Normal rabbit IgG (2729 S, Cell Signaling Technology, USA) was used as a negative control. After primary antibody incubation, sections were washed three times with PBS and incubated with a biotin-labelled anti-rabbit IgG secondary antibody (ZB2010, Zhongshan Golden Bridge, China) for 40 min, followed by incubation with a streptavidin–horseradish peroxidase complex (ZB-2404, Zhongshan Golden Bridge, China) for an additional 40 min. Immunoreactivity was visualised using a 3,3’-diaminobenzidine (DAB) detection kit (ZLI-9018, Zhongshan Golden Bridge, China), and nuclei were counterstained with haematoxylin.

Immunohistochemical staining was independently evaluated by two experienced pathologists blinded to the clinical information. Staining scores were calculated based on the staining intensity and the proportion of positively stained cells. The final immunoreactivity score was used for statistical comparison between BLCA and normal bladder tissues.

### Statistical analysis

All statistical analyses were performed using R software (version 4.1.3; https://www.r-project.org/). For comparisons between two groups, Student’s t-test was applied when data followed a normal distribution, whereas the Wilcoxon rank-sum test was used for non-normally distributed data. Differences in immunohistochemical staining scores between BLCA and normal bladder tissues were analysed using the Wilcoxon rank-sum test.

Overall survival differences between patient groups were evaluated using Kaplan–Meier survival analysis, and statistical significance was assessed with the log-rank test. Univariate and multivariate Cox proportional hazards regression analyses were conducted to identify independent prognostic factors. LASSO Cox regression was performed to construct the prognostic risk model. Time-dependent receiver operating characteristic (ROC) curves were generated using the timeROC package to assess the predictive performance of the model at 1, 3, and 5 years. All statistical tests were two-sided, and *P* < 0.05 was considered statistically significant.

## Results

### Identification of cell types and functional enrichment analysis of marker genes in BLCA

**Fig. 1:**
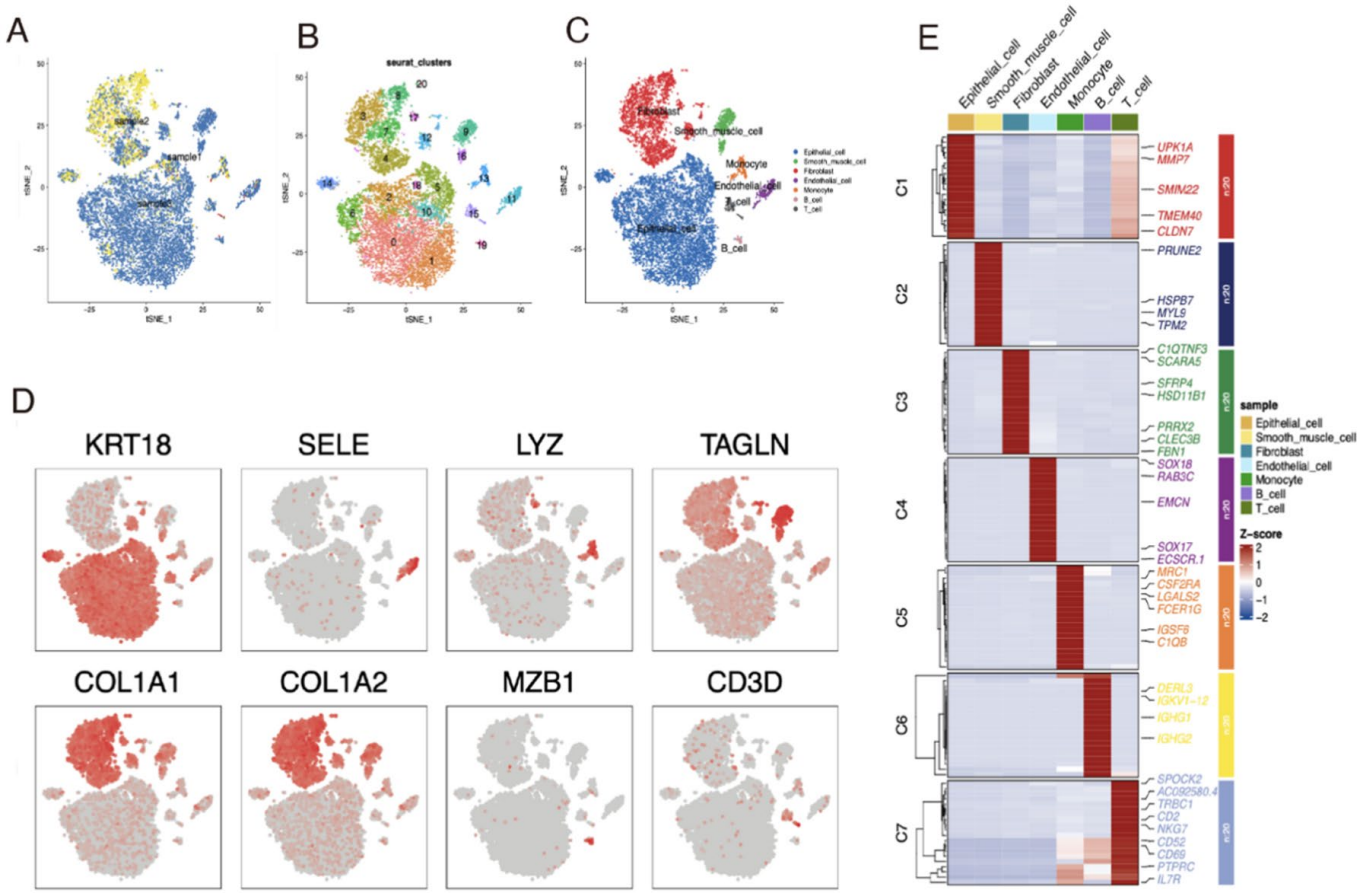
Single-cell transcriptomic analysis of BLCA adjacent to cancer sample. (**A**) t-SNE plot displays transcriptional profiles of individual cells. (**B**) Cells divided into 21 clusters via t-SNE dimensionality reduction using principal component analysis and t-SNE algorithms. (**C**) t-SNE plot with annotated cell types based on cluster-specific gene expression patterns. (**D**) Feature plots for selected marker genes. (**E**) Heatmap shows z-score normalised expression levels of marker genes across clusters, with red indicating high expression and blue low expression.

The scRNA-seq analysis revealed the heterogeneous cell populations present in a tissue sample, as illustrated in the t-distributed stochastic neighbour embedding (t-SNE) plots (Fig. 1A). Subsequent *Seurat* clustering identified 21 distinct groups, each characterised by specific cell type markers (Fig. 1B). Specifically, cell types were identified using the following markers: *KRT18* for epithelial cells, *SELE* for endothelial cells, *LYZ* for monocytes, *TAGLN* for smooth muscle cells, *COL1A1* and *COL1A2* for fibroblasts, *MZB1* for B cells, and *CD3D* for T cells, as shown in Fig. 1C. The t-SNE plots of these markers displayed distinct localisation within the tissue, corresponding to the identified cell clusters (Fig. 1D). Furthermore, the *FindMarkers* function was used to identify the top 50 distinct markers for individual cell clusters. These markers, which serve as indicators of the cell types within each cluster, were visualised in a heatmap to illustrate the unique gene expression profiles associated with each group (Fig. 1E). Supplementary Fig. 2 presents the results of functional enrichment analysis performed on the marker genes within each cell group.

### Analysis of specific regulation of immune cell types

**Fig. 2:**
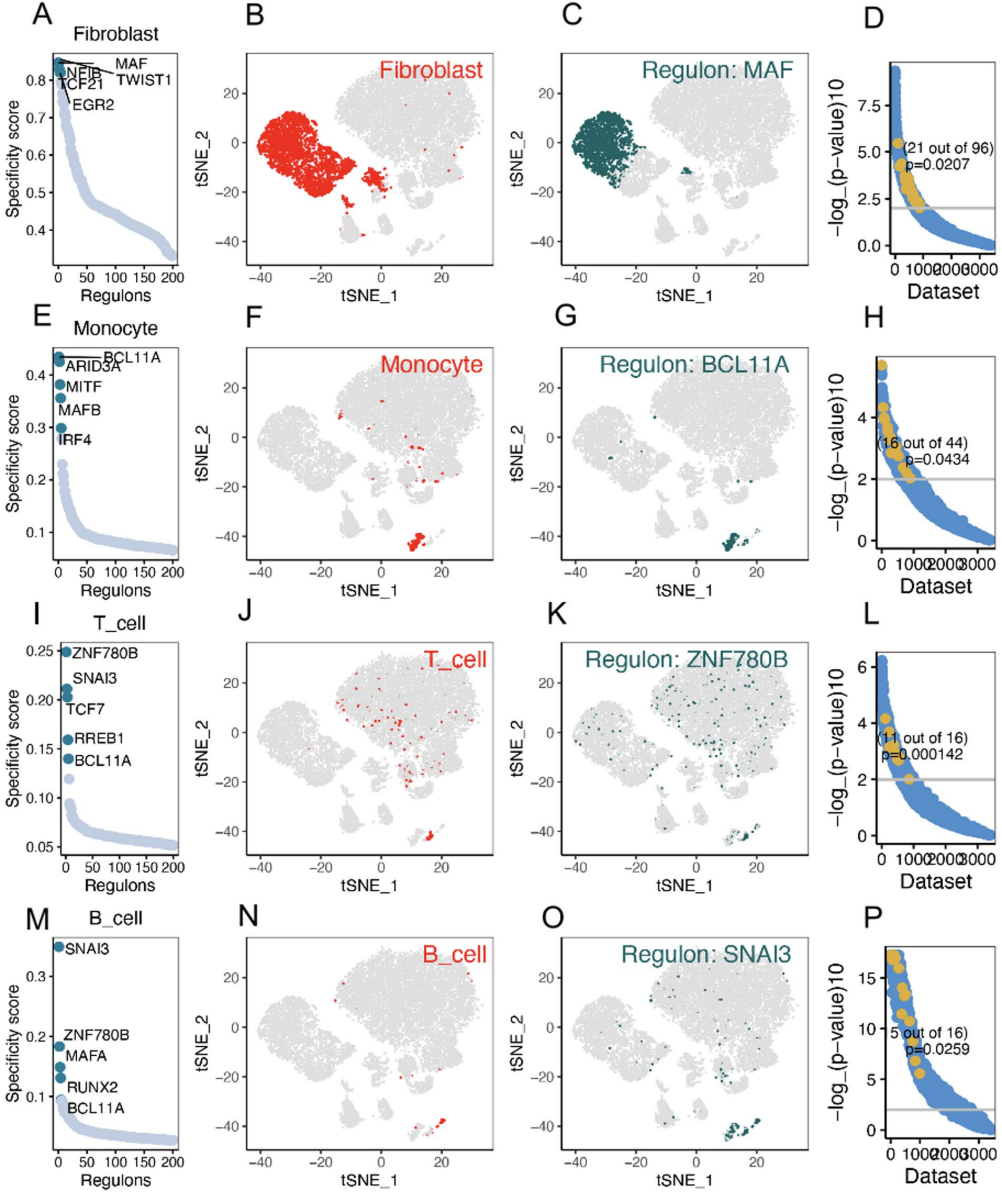
Analysis of cell type-specific regulation in patients with BLCA. (**A**–**D**) Fibroblasts: (**A**) The regulation of fibroblasts was ranked according to the regulation specificity score. (**B**) Fibroblasts are marked by red dots on the t-SNE diagram. (**C**) The expression values of the genes with the highest regulatory activity score are displayed on the t-SNE diagram. (**D**) SEEK analysis was used to identify the co-expression results of the highest regulatory genes in different GEO datasets. The X-axis represents the different datasets, and the Y-axis represents the co-expression significance of the target gene in each dataset. Datasets with significant correlation (*P* < 0.05) are highlighted with yellow dots. (**E**–**H**) Same as (A–D), but applied to monocytes. (**I**–**L**) Same as (A–D), but applied to T cells. (**M**–**P**) Same as (A–D), but applied to B cells.

Our network analysis identified several transcription factors with relatively high regulation specificity in BLCA fibroblasts, including MAF, TWIST1, NFIB, TCF21, and EGR2 (Fig. 2A). Visualization of regulon activity on the t-SNE map showed that MAF regulon activity was predominantly enriched within the fibroblast cluster, indicating a fibroblast-associated regulatory pattern (Fig. 2B,C). To further support this observation, SEEK analysis identified statistically significant co-expression of MAF with cancer-associated fibroblast marker genes across multiple independent GEO datasets (Fisher’s exact test, *P* = 0.0207) (Fig. 2D). In BLCA monocytes, BCL11A, ARID3A, MITF, MAFB, and IRF4 were identified as transcription factors with relatively high regulation specificity (Fig. 2E). Among these, BCL11A displayed a distinct regulon activity pattern in monocytes in the t-SNE space (Fig. 2F,G). SEEK analysis also indicated statistically significant co-expression of BCL11A across GEO datasets (Fisher’s exact test, *P* = 0.0434) (Fig. 2H). Additional cell type–specific regulatory features are presented in Fig. 4I–P.

**Fig. 3:**
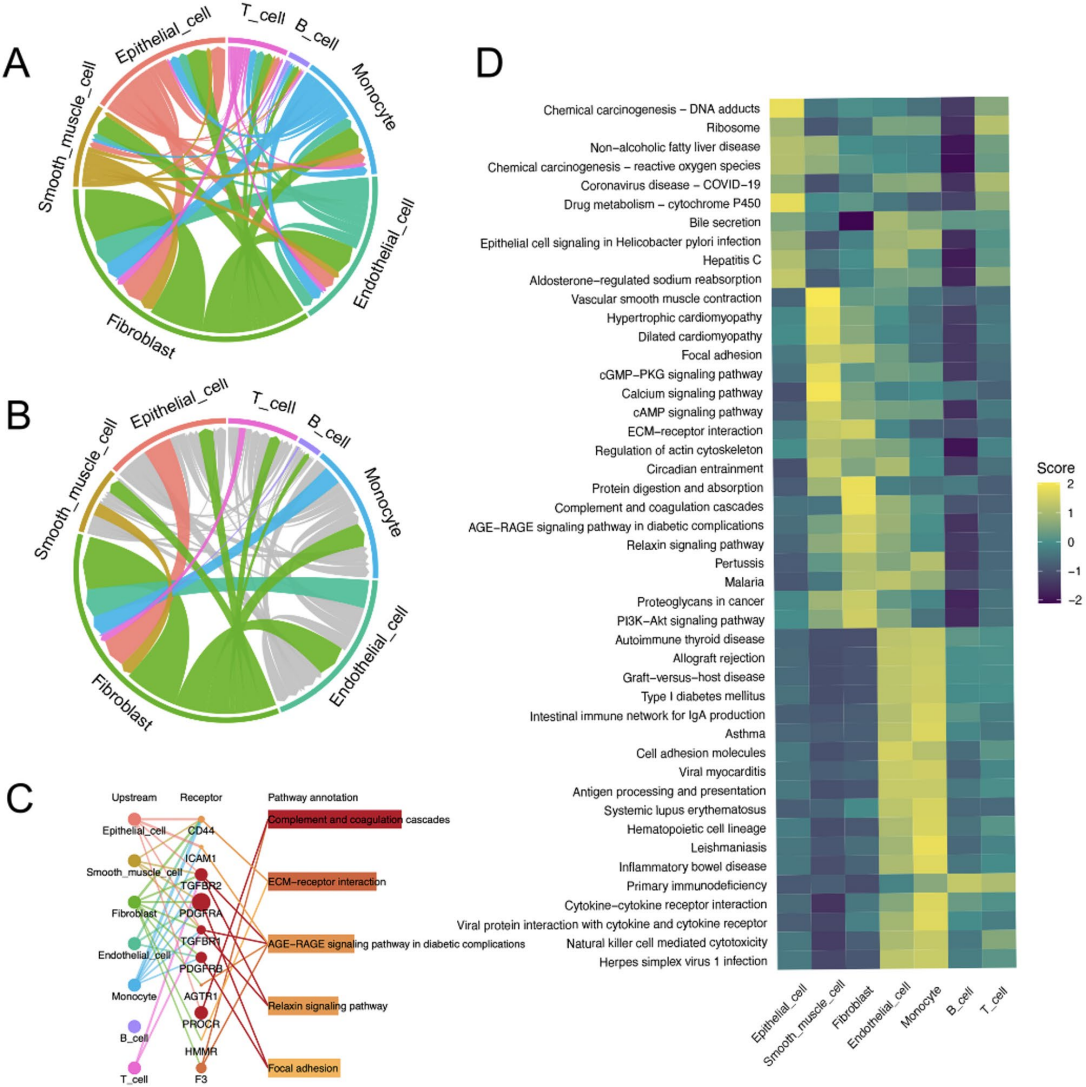
Pathway analysis of BLCA intercellular communication and cell-specific signalling via *CommPath*. (**A**) Circular chord diagram illustrating the relative frequency of intercellular signalling interactions among BLCA microenvironment cell types. (**B**) Similar diagram focusing on fibroblast interactions. (**C**) Network diagram highlighting fibroblast-specific pathways and their interconnections. (**D**) Heatmap summarising pathway activity scores across cell types, with colour intensity indicating activity level.

### Intercellular communication network in BLCA revealed by *CommPath* analysis

Figure 3A presents a circular chord diagram quantifying the signalling interactions among BLCA cells, with each segment representing a specific cell type. The ribbons within the diagram indicate the frequency of signalling interactions, while their width reflects the strength of these interactions. In Fig. 3B, the focus is narrowed to fibroblast communications within the tumour stroma, using a similar diagram to emphasise the importance of their signalling within this microenvironment. Figure 3C shows a network diagram highlighting pathways centred around fibroblasts. Nodes represent different cell types, while edges depict direct interactions within pathways such as the complement and coagulation cascades, and the AGE–RAGE signalling pathway. The heatmap in Fig. 3D illustrates pathway activity scores across various cell types, with columns representing cell types, rows representing pathways, and colours indicating the range of activity scores from high (yellow) to low (purple). This analysis reveals a comprehensive BLCA signalling network, highlighting cell-specific pathways as potential therapeutic targets. Particularly notable are the fibroblast pathways and interactions, which underscore their pivotal role in BLCA progression and the potential of targeting the TME in cancer treatment strategies.

### Characterization of metabolic module flux in BLCA fibroblasts

**Fig. 4:**
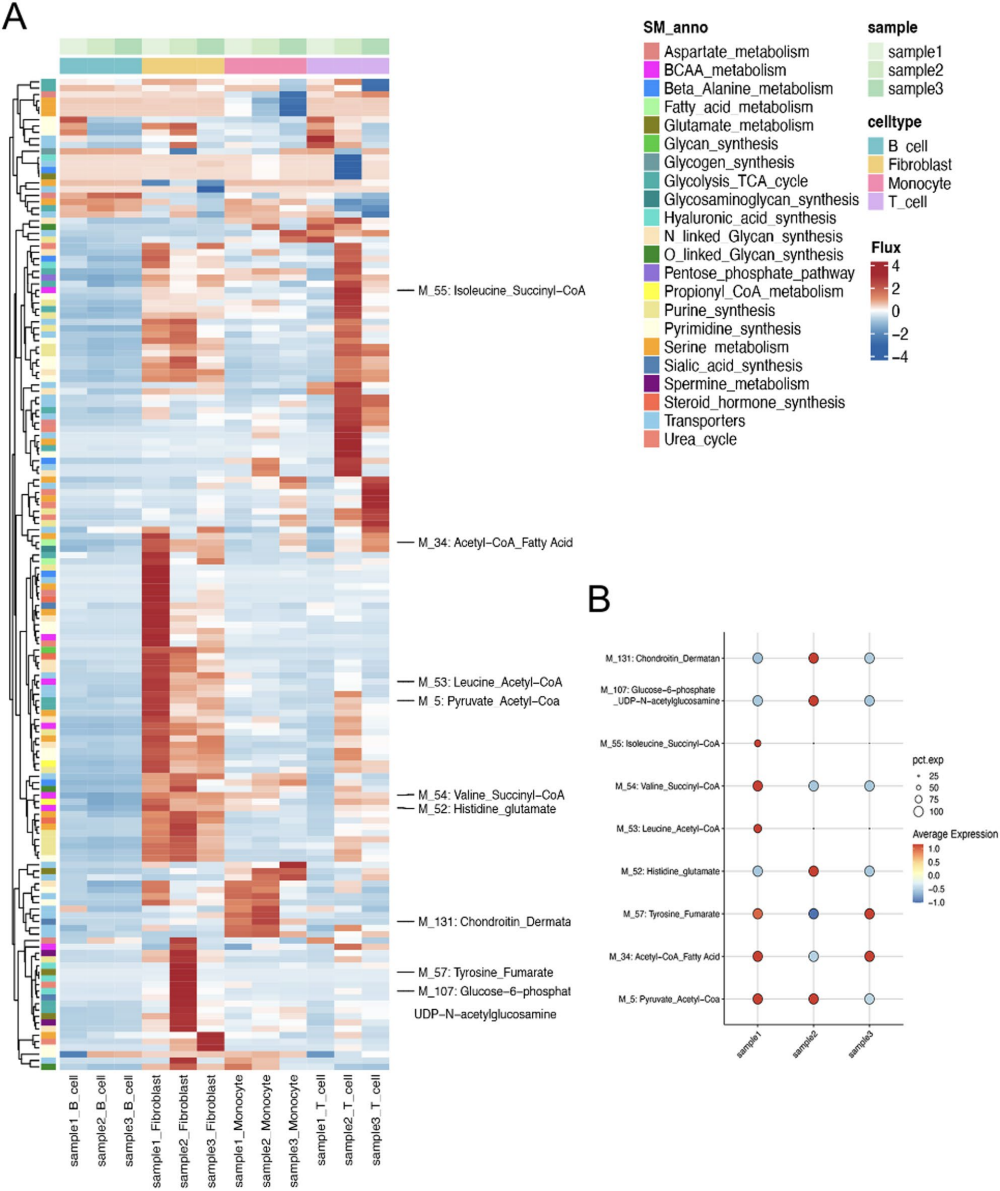
scFEA depicting metabolic pathway activity across diverse cell types. (**A**) Heatmap displaying pathway flux across cells, with rows indicating pathway flux and columns representing single cells by type and origin. Red denotes higher flux; blue denotes lower. Left-side clustering groups pathways with similar activity profiles. (**B**) Summary plot showing variability of selected pathways within cell types. Points represent pathways, with colour intensity reflecting average expression level.

Figure 4 illustrates the metabolic profile of fibroblasts in the BLCA microenvironment, using scFEA to measure metabolic module activity. In Fig. 4A, we observe the flux intensities for fibroblast-specific metabolic pathways: M_5, representing the pyruvate to acetyl-CoA transition, essential for fuelling the Krebs cycle; M_34, acetyl-CoA to fatty acid synthesis, a cornerstone of lipid metabolism and membrane formation; M_57, tyrosine to fumarate catabolism, linking amino acid degradation to the TCA cycle; and M_107, glucose-6-phosphate to UDP-N-acetylglucosamine, a precursor for glycosylation processes. The heatmap displays a range of flux activities across these modules, with differences in colour intensity reflecting varying metabolic demands or states within the fibroblast population. Figure 4B presents a bubble plot showing the relative activities of these metabolic modules across three distinct samples. Examining fluctuations in these module activities using scFEA may reveal functional states of fibroblasts and their involvement in disease progression.

**Fig. 5:**
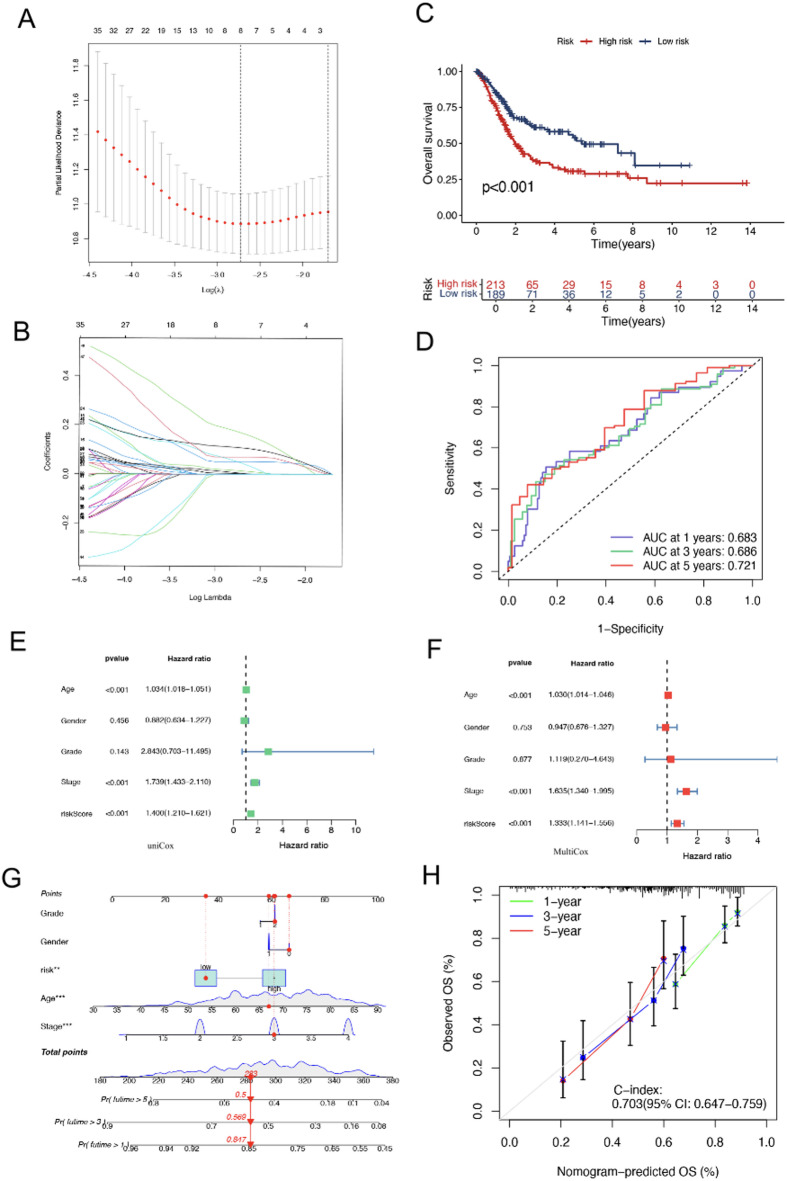
Analysis of risk scores for survival prediction. (**A**) Optimal feature selection using LASSO coefficients. (**B**) Lambda tuning in the LASSO model. (**C**) Survival stratification based on risk groups. (**D**) Predictive accuracy assessed via receiver operating characteristic curves. (**E**) Univariate Cox analysis of clinical characteristics. (**F**) Multivariate Cox model adjusting for covariates. (**G**) Nomogram predicting survival probabilities. (**H**) Calibration plot evaluating nomogram prediction accuracy.

### Prognostic risk assessment and survival prediction in BLCA

Figure 5 presents a comprehensive analysis of prognostic risk scores and their association with overall survival in patients with BLCA. A total of 71 candidate genes were initially identified by intersecting fibroblast marker genes derived from single-cell analysis with prognostic-related genes from the TCGA-BLCA cohort. Based on the fibroblast-related regulon analysis, three extracellular matrix–associated genes, *FBN1*, *PID1*, and *PRELP* were selected for subsequent prognostic modelling. As shown in Fig. 5A, LASSO coefficient profiles were plotted against log(lambda), highlighting features with non-zero coefficients at the optimal lambda value. Figure 5B illustrates the selection of the optimal lambda based on ten-fold cross-validation, with the vertical dashed line indicating the value corresponding to the minimum cross-validation error.

Subsequently, a risk score was constructed:

$$\begin{aligned} {\text{Risk score }} &= (0.0{\text{954 }} \times FBN1\;{\text{expression }}) + {\text{ }}\left( {0.{\text{1315 }} \times PID1\;{\text{expression }}} \right) \\ & \quad + {\text{ }}\left( {0.0{\text{264 }} \times PRELP\;{\mathrm{expression}}} \right). \end{aligned}$$

In Fig. 5C, Kaplan–Meier survival curves are used to stratify patients into high- and low-risk groups based on their risk scores. The survival probability for the high-risk group is notably lower than that of the low-risk group, as evidenced by the significant divergence of the curves (*P* < 0.001), indicating reduced overall survival for the high-risk group throughout the study period. Figure 5D presents time-dependent receiver operating characteristic curves evaluating the predictive efficacy of the risk score at various intervals. The area under the curve (AUC) values for 1-year, 3-year, and 5-year survival are 0.683, 0.686, and 0.721, respectively. Figure 5E and F depicts forest plots showcasing the results of univariable and multivariable Cox regression analyses, respectively, elucidating the impact of the risk score in combination with additional clinical factors on patient survival. The nomogram shown in Fig. 5G integrates these variables to forecast the likelihood of survival at 1, 3, and 5 years, providing a valuable tool for clinical prognostication. Finally, the calibration plot in Fig. 5H for the nomogram demonstrates the alignment between predicted and actual survival probabilities, with closer alignment to the 45-degree line indicating better calibration. Through these assessments, Fig. 5 confirms the reliability of the risk scores in predicting survival, as evidenced by the strong predictive performance indicated by the AUC values—particularly notable at the 5-year interval. Suggesting that the model has potential prognostic value, though further validation is needed.

### Risk stratification and survival analysis based on gene expression profiles

**Fig. 6:**
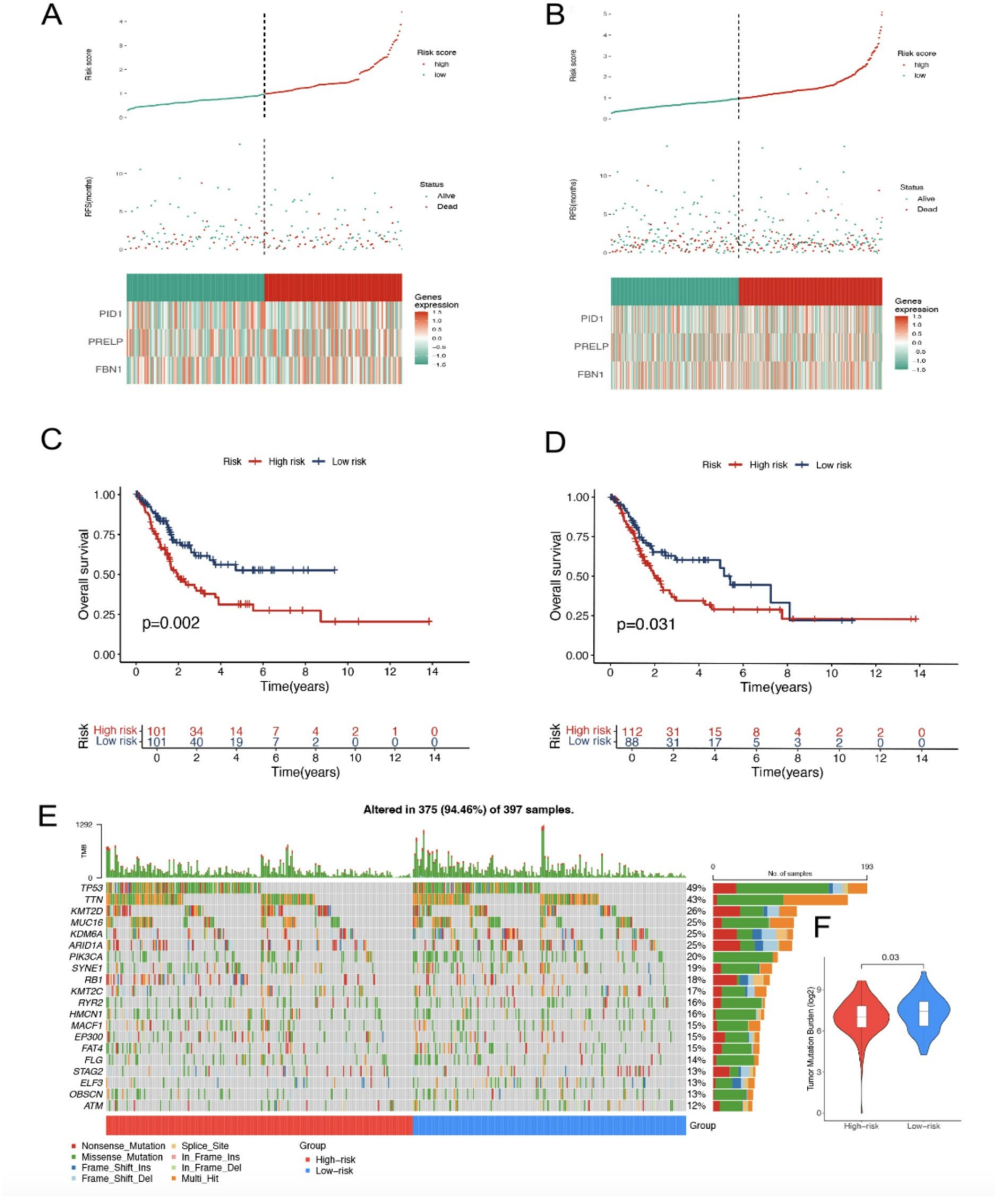
Risk score impact on survival in training and test cohorts. (**A**) Risk scores, survival status, and gene expression in the training group. (**B**) Risk distribution and survival outcomes in the test group. (**C**,**D**) Kaplan–Meier survival curves for training and test cohorts. (**E**) Genetic alterations in high- and low-risk groups. (**F**) TMB comparison by risk category.

Figure 6 presents an integrated analysis of risk scores derived from gene expression profiles and their prognostic relevance in both the training and test patient cohorts. In Fig. 6A, the distribution of risk scores in the training cohort is shown, categorising patients into high- and low-risk groups based on their gene expression patterns. The classification is guided by their gene expression signatures, with a vertical dotted line indicating the threshold used for risk stratification.

The relationship between patient survival status is illustrated, showing a clear separation between living and deceased patients across the various risk categories. Figure 6B replicates this analysis in the test cohort, confirming the consistency of risk stratification when applied to an independent dataset. The risk score threshold effectively distinguishes between high- and low-risk patients, aligning with differences in survival outcomes. In Fig. 6C, Kaplan–Meier survival analysis of the training cohort demonstrates a significant difference in overall survival rates between the high- and low-risk groups (*P* = 0.002). Patients with elevated risk scores exhibit markedly reduced survival throughout the study period. This finding is further supported by the Kaplan–Meier survival curves shown in Fig. 6D for the test cohort, which confirm the prognostic value of risk stratification, with a similarly notable difference in survival probabilities (*P* = 0.031), consistent with the observations from the training cohort. Additionally, Fig. 6E illustrates the mutation landscape across both cohorts, highlighting the distribution of mutations in key cancer-associated genes. This oncoprint provides a visual representation of the mutational status of genes such as *TP53*, *TTN*, and *MUC16*, offering valuable insights into the potential genetic contributors to the calculated risk scores. Figure 6F presents a comparison of the TMB between high- and low-risk groups. The high-risk group exhibits a significantly lower TMB (*P* = 0.03), indicating that reduced mutational burden is associated with increased patient risk within this context.

**Fig. 7:**
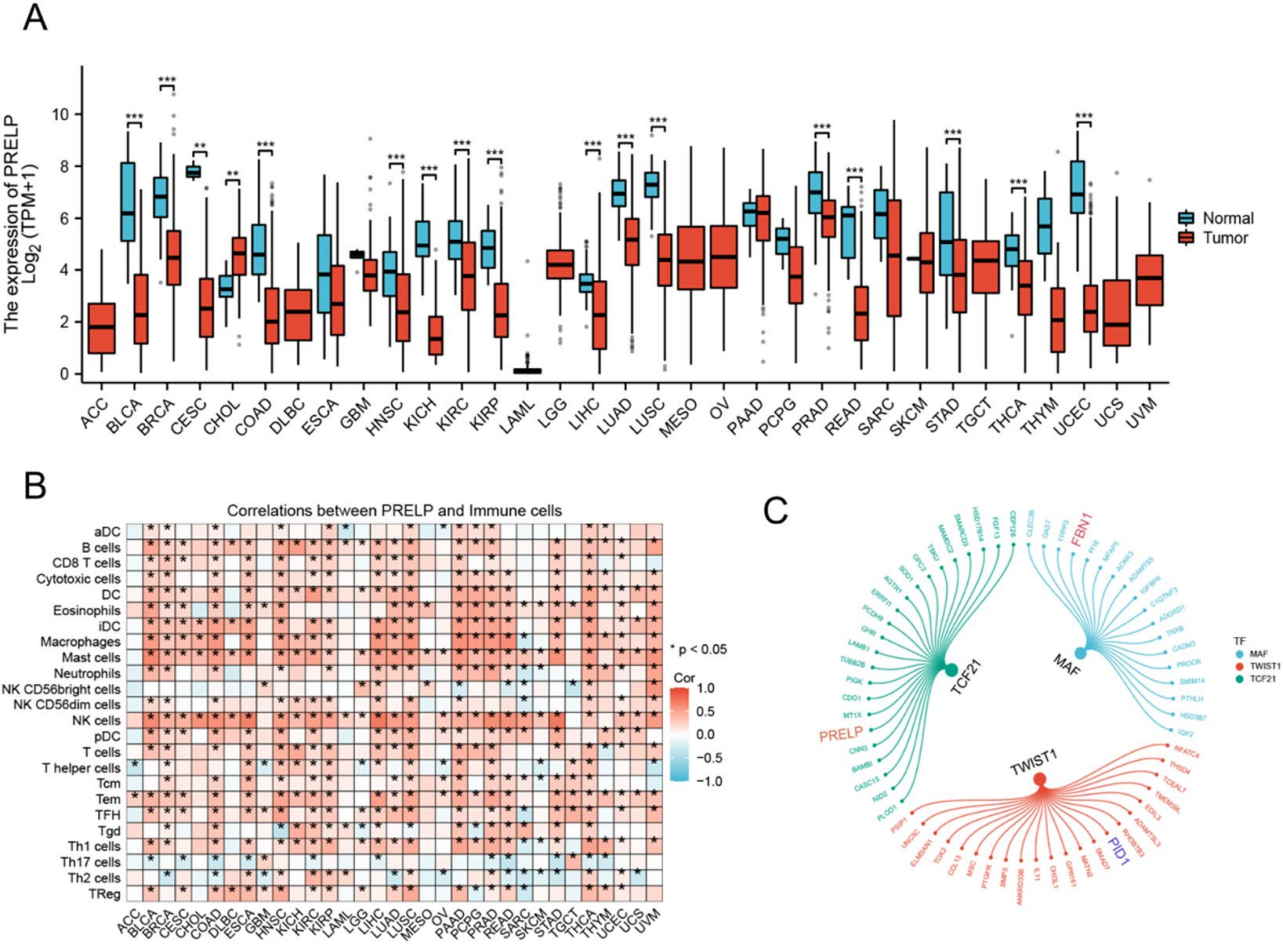
Analysis of *PRELP* expression and immune cell interactions in cancer. (**A**) Boxplots displaying *PRELP* expression across multiple cancer types compared with normal samples. (**B**) Heatmap showing correlations between *PRELP* expression and various immune cell types across cancers. (**C**) Network diagram illustrating the relationships between TFs and their target genes in fibroblasts.

### Expression of *PRELP* in cancer and its correlation with immune infiltration

Figure 7 illustrates the expression patterns of *PRELP* across multiple cancer types and its association with immune cell infiltration. Specifically, Fig. 7A presents boxplots comparing *PRELP* expression levels between normal and tumour tissues across a wide range of cancers. Overall, *PRELP* expression was generally lower in tumour tissues compared with corresponding normal tissues in most cancer types, although the magnitude and direction of this difference varied across cancers. Cholangiocarcinoma represented a notable exception, exhibiting relatively higher *PRELP* expression in tumour tissues. Statistical significance, as indicated by asterisks, reflects differences between tumour and normal samples rather than a uniform direction of change. Figure 7B displays a heatmap illustrating the correlations between *PRELP* expression and the infiltration of various immune cell populations across different cancers. The colour scale denotes correlation coefficients, with red indicating positive correlations and blue indicating negative correlations. *PRELP* expression showed predominantly positive associations with activated dendritic cells in several cancer types, while negative correlations with regulatory T cells were observed in a subset of cancers, highlighting the heterogeneity of the tumour immune microenvironment. Supplementary Figs. 3 and 4 present the pancancer expression profiles of *FBN1* and *PID1*, respectively. Based on fibroblast-associated regulons identified in the SCENIC analysis, a regulatory network was constructed to visualise the relationships between transcription factors and their downstream target genes (Fig. 7C). Within this network, *FBN1* was identified as a putative target of MAF, *PID1* as a putative target of TWIST1, and *PRELP* as a putative target of TCF21. These transcriptional associations suggest potential regulatory mechanisms through which fibroblast-related transcription factors may influence extracellular matrix organisation and fibroblastic activity within the tumour microenvironment.

### Differential gene expression between BLCA and normal bladder tissue

**Fig. 8:**
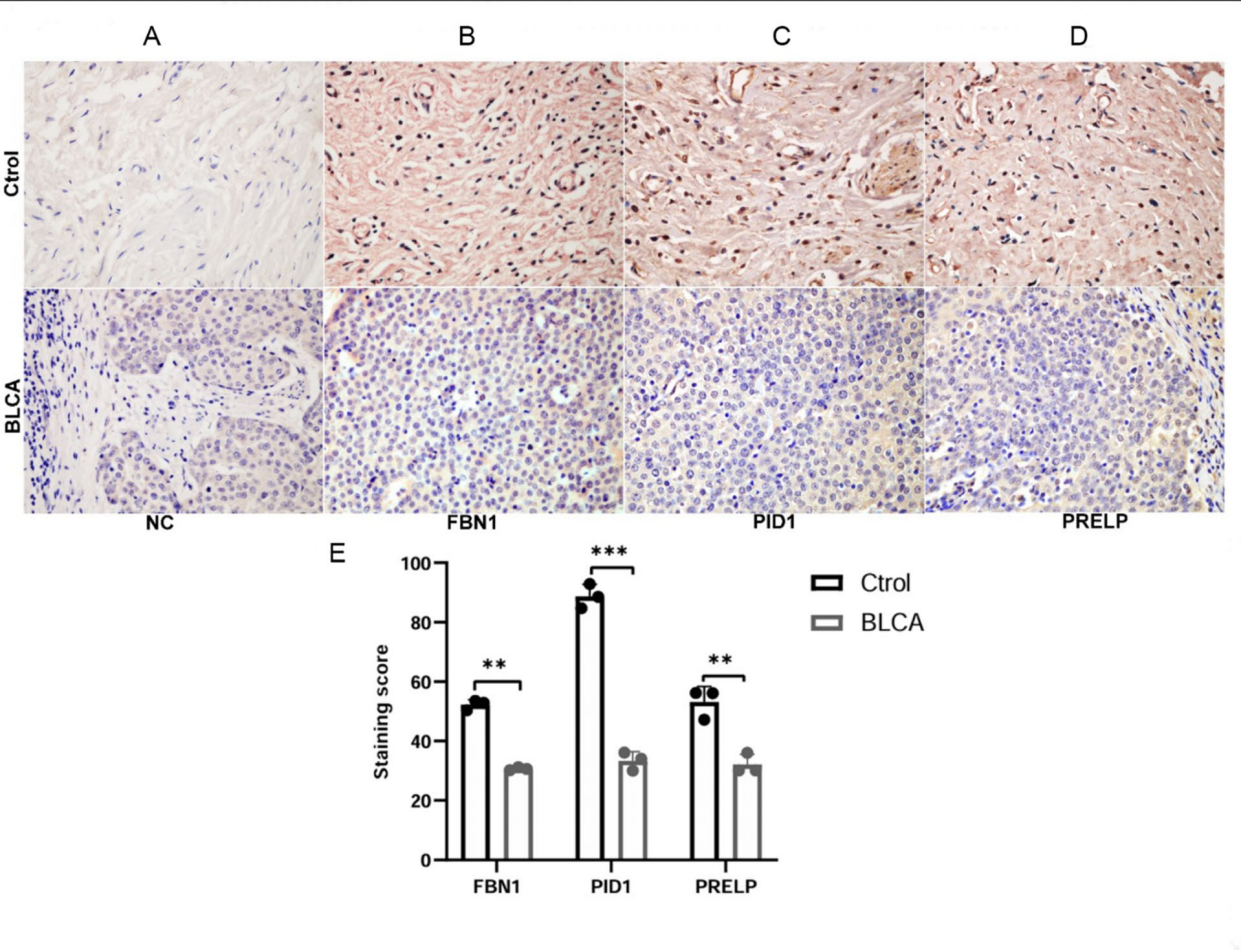
Expression patterns of *FBN1*, *PID1*, and *PRELP* in BLCA and normal bladder tissues. (**A**) Negative control (NC). (**B**–**D**) Representative immunohistochemical staining images of *FBN1*, *PID1*, and *PRELP* in normal bladder tissues (upper panels) and BLCA tissues (lower panels). Compared with normal bladder tissues, BLCA tissues exhibited reduced overall staining intensity of *FBN1*, *PID1*, and *PRELP*. (**E**) Quantification of immunohistochemical staining scores. Data are presented as mean ± SD. Ctrol, control; BLCA, bladder cancer. Scale bar, 20 μm.

To investigate alterations in the expression of ECM-related proteins in BLCA, immunohistochemical analyses were performed on BLCA tissues and matched normal bladder tissues. As shown in Fig. 8A–D, the overall staining intensity of *FBN1*, *PID1*, and *PRELP* was reduced in BLCA tissues compared with normal bladder tissues. Consistent with the representative images, quantitative analysis of immunohistochemical staining scores further confirmed a significant reduction of *FBN1*, *PID1*, and *PRELP* staining in BLCA tissues (Fig. 8E).

## Discussion

The rapid development of single-cell sequencing has offered a unique perspective on the cellular diversity within the TME, shedding light on the intricate interactions that drive cancer development and influence treatment outcomes. This study explores the specific involvement of fibroblasts in BLCA within this complex milieu^18,27^.

The identification of active TFs, MAF, TWIST1, and TCF21 within the BLCA microenvironment underscores a critical aspect of TME regulation. The recognition of MAF as a key factor in CAFs is particularly noteworthy, given its established role in immune regulation and its potential involvement in oncogenesis^28^. TWIST1, known for its role in epithelial–mesenchymal transition, adds further complexity by promoting tumour invasion and metastasis^29^. The presence of TCF21, associated with differentiation pathways, suggests a sophisticated regulatory network that shapes fibroblast phenotypes within BLCA^30^. Our SCENIC analysis reinforces the importance of these TFs by linking them to specific gene targets, including *FBN1*, *PID1*, and *PRELP*.

In BLCA and other solid tumours, CAFs play a significant role in ECM formation and in modulating the immune microenvironment, thereby influencing cancer progression and therapeutic responses^31,32^. Within this context, fibrillin-1, encoded by the *FBN1* gene, serves both structural and regulatory functions.

*Fibrillin-1*, a key component of the ECM, influences the behaviour of CAFs and the TME by interacting with critical signalling molecules such as transforming growth factor-beta and bone morphogenetic proteins^33^. Elevated expression of *FBN1* has been observed in various cancer types, suggesting its potential utility as a biomarker—particularly in fibrotic and cancerous tissues—thereby implicating it in aberrant ECM remodelling processes^34^.

Additionally, *PID1* and *PRELP* have been identified as key players in cancer progression through their roles in modulating ECM functions^35^. *PID1* is known to regulate apoptosis and cell survival pathways, thereby influencing cancer cell resistance to treatment, particularly in hepatocellular carcinoma. Its involvement in the regulation of apoptosis is mediated through critical signalling pathways such as AKT/Raf-1^36^.

*PRELP* plays a crucial role in cell–cell adhesion and ECM integrity, as well as in the regulation of epithelial–mesenchymal transition, thereby influencing migration and invasion in cancers such as hepatocellular carcinoma^37^. The diverse functions of *PRELP* in modulating structural integrity and cellular transitions have a substantial impact on tumour progression and metastasis^38^. The expression levels of *FBN1*, *PID1*, and *PRELP* for the ECM status and its interaction with cancer progression and immune response, offering prognostic value. These correlations—specifically through modulation of the TME or signalling pathways involved in cellular proliferation and differentiation. may serve as candidate biomarkers highlight possible avenues for therapeutic investigation.

The incorporation of *FBN1*, *PID1*, and *PRELP* into our prognostic model for BLCA underscores their importance in predicting 5-year survival rates, as demonstrated by a high AUC value that reflects the model’s strong capability to stratify patients into distinct risk groups. Although *FBN1*, *PID1*, and *PRELP* were downregulated in BLCA compared with normal tissues, higher expression of these genes within tumor samples was associated with poorer overall survival. This likely reflects increased stromal fibroblast activity and extracellular matrix remodeling in high-risk tumors rather than tumor cell–intrinsic expression. Taken together, these findings suggest that the prognostic value of this model is primarily driven by stromal reprogramming within the tumor microenvironment, representing a preliminary approach that requires independent validation.

Metabolic flux analysis of BLCA was conducted using scFEA. The findings related to metabolic pathways—such as the conversion of pyruvate to acetyl-CoA and the synthesis of fatty acids from acetyl-CoA—highlight the metabolic adaptability of cancer cells^39,40^. These pathways support the energetic and biosynthetic demands of rapidly dividing cancer cells and may also contribute to the creation of an immunosuppressive environment that undermines treatment efficacy^41^. Our research aligns with the concept of metabolic cooperation between stromal and cancer cells, pointing to a promising direction for therapeutic strategies^42^. Furthermore, the varying levels of metabolic flux observed across distinct BLCA samples underscore the functional diversity within CAF populations. This metabolic heterogeneity may indicate or influence the functional polarisation of CAFs, with roles ranging from tumour suppression to tumour promotion^43^.

Lower TMB was observed in the high-risk prognostic group, with previous studies indicating a potential reduction in the efficacy of immunotherapy in cases of a lower TMB^44,45^. This finding may suggest diminished immunogenicity or reduced effectiveness of immune checkpoint blockade^46^. The correlation between the TMB and risk categories underscores the importance of prognostic models within the evolving landscape of cancer treatment strategies involving immune checkpoint inhibitors^44,47^.

Looking ahead, it is crucial to investigate the causal connections between TFs and metabolic pathways—an ongoing and evolving area of research. Our computational pathway analysis and single-cell functional enrichment analysis suggest that the TME is a complex, adaptive system shaped by cellular signalling, metabolic changes, and treatment effects. The decreased expression levels of *FBN1*, *PID1*, and *PRELP* in tumour tissues relative to normal tissues across various cancer types further support the hypothesis that these genes may exert tumour-suppressive functions. Moreover, the association between *PRELP* expression and immune infiltration suggests a potentially novel immune-modulatory pathway, thereby deepening our understanding of the immune microenvironment in BLCA.

This study has several limitations. The immunohistochemistry validation was performed on a small cohort (*n* = 3), which restricts generalizability. Transcriptomic analyses relied on a single public dataset, which may introduce bias. Our prognostic model was not validated in an independent cohort, and the associations reported remain correlative without mechanistic validation. Future studies with larger, multi-center cohorts and experimental validation are required.

In summary, our comprehensive investigation of the BLCA TME—integrating transcriptional regulation, metabolic pathways, and prognostic modelling—provides a multifaceted perspective on the role of fibroblasts in disease progression. While this study advances current understanding of fibroblast-centred regulatory and prognostic mechanisms in BLCA, it also has several limitations, including reliance on computational analyses, a small immunohistochemistry validation cohort, and the absence of independent external validation. Therefore, further experimental studies, including animal models and larger multi-centre cohorts, are required to validate these findings. Future research should focus on translating these molecular insights into clinically actionable strategies targeting cancer-associated fibroblasts and the tumour microenvironment to improve patient prognosis.

## Conclusion

In this study, we integrated single-cell and bulk transcriptomic analyses to characterise the immune microenvironment of bladder cancer, with a focus on fibroblast-associated regulatory and metabolic features. Using SCENIC and multi-omics approaches, key fibroblast-related transcription factors and extracellular matrix–associated genes, including *FBN1*, *PID1*, and *PRELP* were identified. Based on these fibroblast-derived molecular features, a prognostic risk model was constructed that effectively stratified patients with BLCA into distinct risk groups and showed stable predictive performance for overall survival. Although further validation in independent cohorts and functional studies is required, our findings highlight the prognostic relevance of fibroblast-centred molecular signatures and their potential value in BLCA.

## Supplementary Information

Below is the link to the electronic supplementary material.

Supplementary Material 1

## Data Availability

RNA sequencing data used in this study are available in the Gene Expression Omnibus (GEO) under accession code GSE129845.

## Abbreviations

BLCA: Bladder cancer
TME: Tumour microenvironment
CAFs: Cancer-associated fibroblasts
TCGA: The Cancer Genome Atlas
GEO: Gene Expression Omnibus
PCA: Principal component analysis
DEGs: Differentially expressed genes
GO: Gene ontology
GRNs: Genetic regulatory networks
RAS: Regulon activity score
SEEK: Search-based exploration of expression
LASSO: Least absolute shrinkage and selection operator
GC: Gastric cancer
ROC: Receiver operating characteristic
MAF: Mutation annotation format
TMB: Tumor mutation burden
GTEx: Genotype-tissue expression
scFEA: Single-cell flux estimation analysis
EMT: Epithelial-mesenchymal transition
ECM: Extracellular matrix
TGF-β: Transforming growth factor-beta
BMPs: Bone morphogenetic proteins
AUC: Area under the curve
FBN1: Fibrillin-1
*PID1*: Phosphotyrosine interaction domain-containing 1
*PRELP*: Proline/arginine-rich end leucine-rich repeat protein
