# Supplementary material for: Single-cell transcriptomics identifies fibroblast associated immune heterogeneity and prognostic signatures in bladder cancer
Source: Sci Rep. 2026 Feb 3;16:7151. doi: 10.1038/s41598-026-38219-x (PMC12920899; doi:10.1038/s41598-026-38219-x)
Supplement: Supplementary file 1 — Supplementary Material 1 [file 41598_2026_38219_MOESM1_ESM.docx]

Supplementary Material

**Supplementary Figures**

**
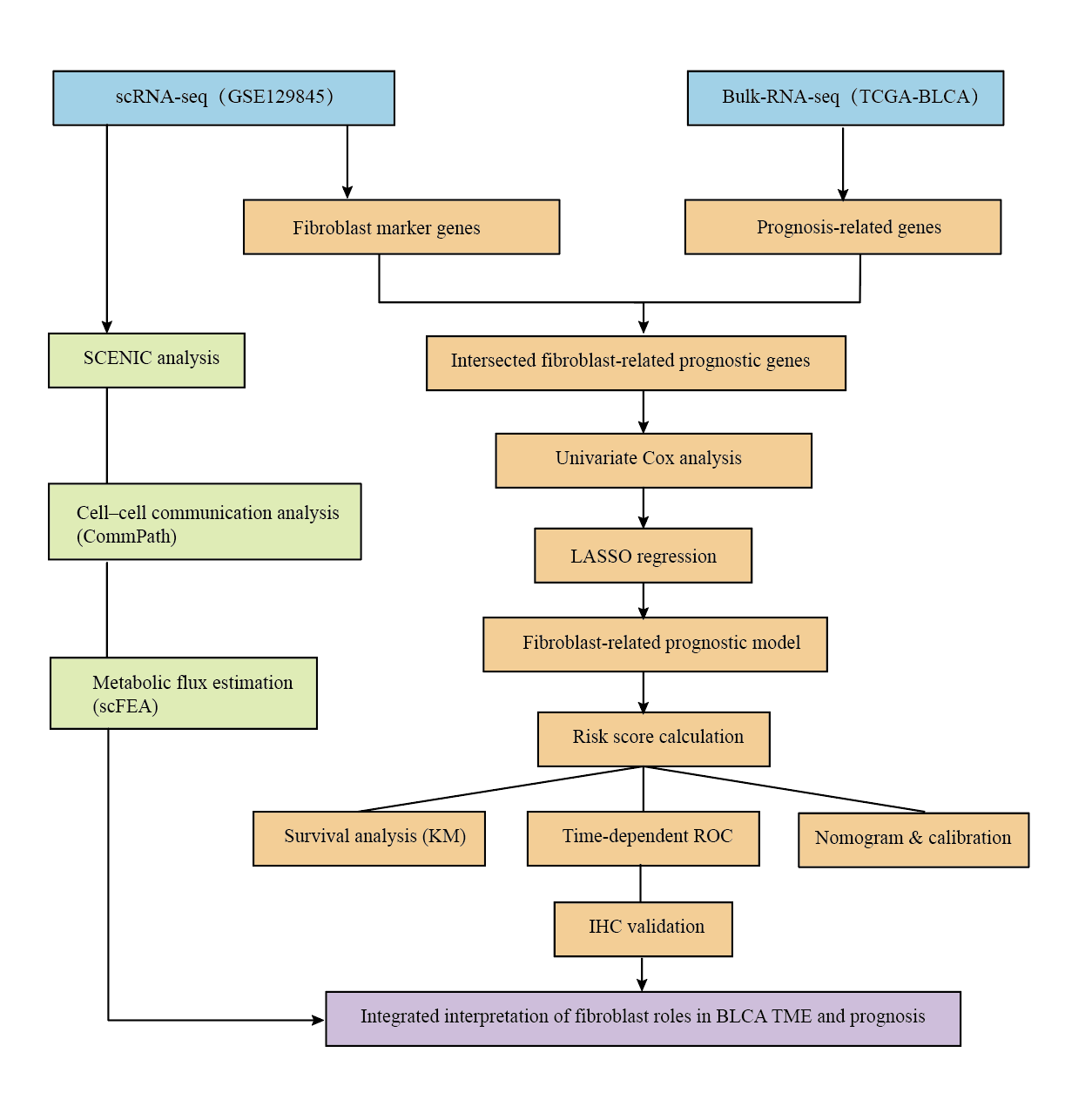
**

**Supplementary Figure 1.** Overview of the analytical workflow integrating single-cell and bulk transcriptomic analyses for fibroblast characterisation and prognostic model construction in bladder cancer.


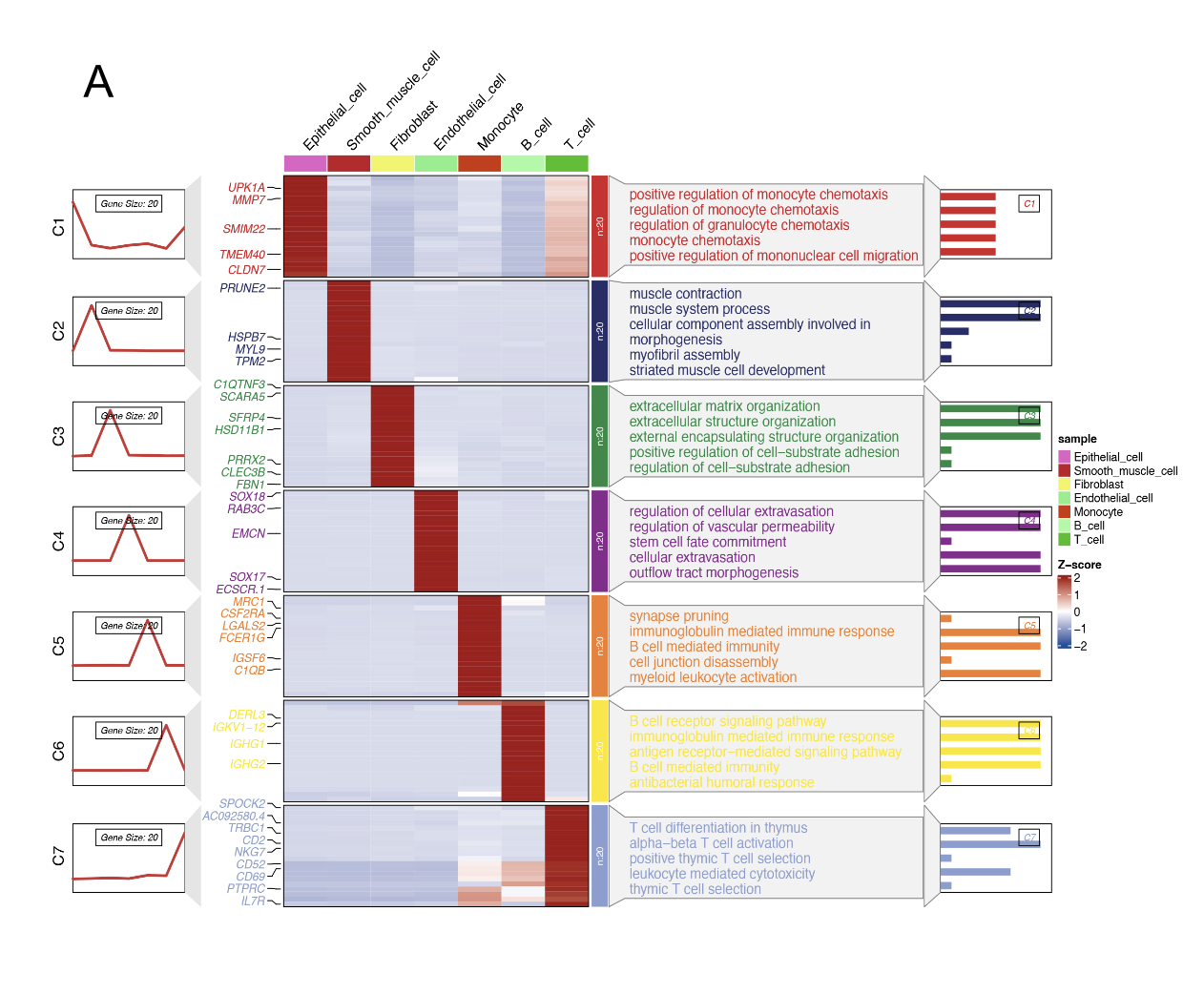


Supplementary Figure 2. Gene expression profiling and functional enrichment analysis across different cell types. The heatmap displays differential gene expression levels in various cell types including epithelial cells, smooth muscle cells, fibroblasts, endothelial cells, monocytes, and T cells. Gene clusters (C1-C7) are identified based on expression patterns specific to these cell types. Each cluster is accompanied by a line graph showing gene count and a box on the right listing the enriched biological functions associated with the marker genes in that cluster.


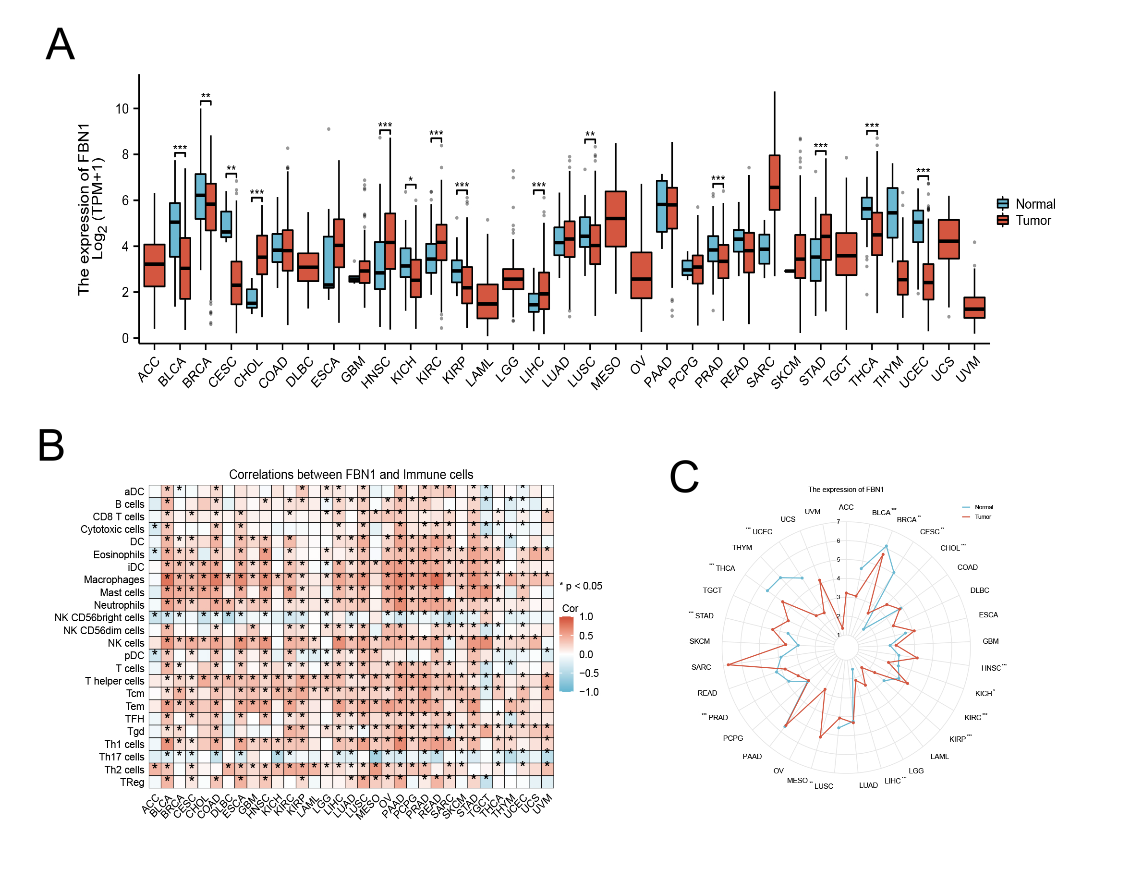


Supplementary Figure 3. Analysis of FBN1 expression and immune cell interactions in cancer. (A) Boxplots displaying FBN1 expression in multiple cancers against normal samples. (B) Heatmap showing correlations of PRELP expression with diverse immune cells across cancer types. (C) Radar chart comparing the expression levels of FBN1between normal and tumor tissues across various cancer types.


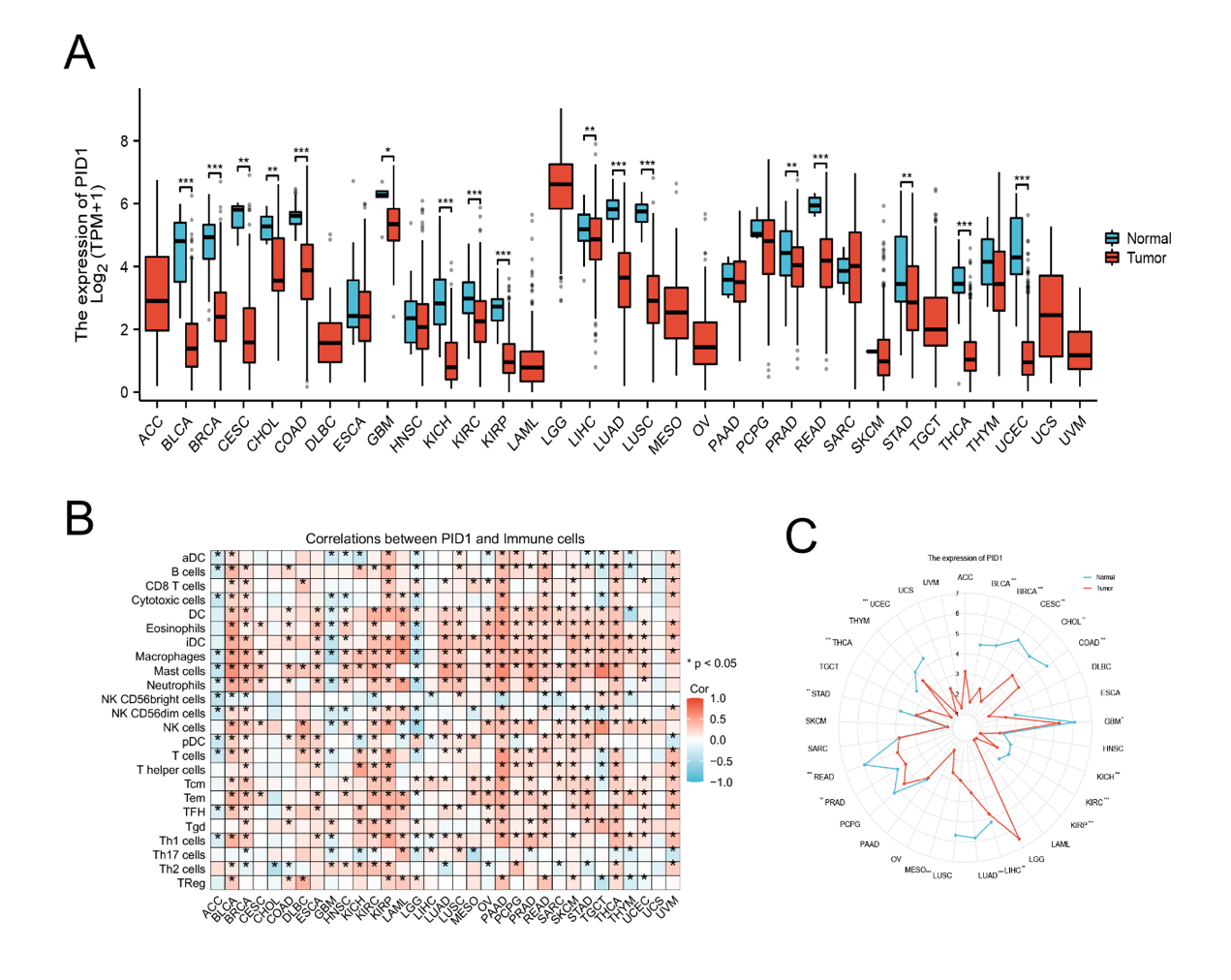


Supplementary Figure 4. Analysis of PID1 expression and immune cell interactions in cancer. (A) Boxplots displaying PID1 expression in multiple cancers against normal samples. (B) Heatmap showing correlations of PRELP expression with diverse immune cells across cancer types. (C) Radar chart comparing the expression levels of P1D1 between normal and tumor tissues across various cancer types.
